# Health Care Industry Insights: Why the Use of Preventive Services Is Still Low

**DOI:** 10.5888/pcd16.180625

**Published:** 2019-03-14

**Authors:** Susan Levine, Erin Malone, Akaki Lekiachvili, Peter Briss

**Affiliations:** 1 Deloitte Consulting, LLP, Atlanta, Georgia; 2 Office of the Medical Director, National Center for Chronic Disease Prevention and Health Promotion, Centers for Disease Control and Prevention, Atlanta, Georgia

Chronic diseases are a tremendous burden to both patients and the health care system. In 2014, 60% of adult Americans had at least one chronic disease or condition, and 42% had multiple diseases (1). Chronic diseases, including heart disease, cancer, chronic lung disease, stroke, Alzheimer’s disease, diabetes, osteoarthritis, and chronic kidney disease, are the leading causes of poor health, long-term disability, and death in the United States (2,3). One-third of all deaths in this country are attributable to heart disease or stroke, and every year, more than 1.7 million people receive a diagnosis of cancer (2). During the past several decades, the prevalence of diabetes increased dramatically; in 2015 more than 29 million Americans had diabetes and another 86 million adults had prediabetes, increasing their chance of developing type 2 diabetes (3). Diabetes increases the risk of developing other chronic diseases, including heart disease, stroke, and hypertension, and is the leading cause of end-stage renal failure (4).

Chronic diseases can profoundly reduce quality of life for patients and for their families, affecting enjoyment of life, family relationships, and finances (5). Working can be difficult for people with chronic diseases: rates of absenteeism are higher and income is often lower among people who have a chronic disease compared with people who do not have one. Functional limitations can be distressing, and depression, which can reduce a patient’s ability to cope with pain and worsen the clinical course of disease, is a common complication (6).

Chronic diseases are also the leading drivers of health care costs in the United States (2). In 2016, total direct costs for health care treatment of chronic diseases were more than $1 trillion, with diabetes, Alzheimer’s, and osteoarthritis being the most expensive (2,7). If lost economic productivity is also considered, the total cost of chronic diseases increases to $3.7 trillion, which is close to one-fifth of the entire US economy (7,8). These costs are expected to increase as the population ages — projections indicate that by 2030, more than 80 million people in the United States will have at least 3 chronic diseases (7).

Clinical preventive strategies are available for many chronic diseases; these strategies include intervening before disease occurs (primary prevention), detecting and treating disease at an early stage (secondary prevention), and managing disease to slow or stop its progression (tertiary prevention). These interventions, combined with lifestyle changes, can substantially reduce the incidence of chronic disease and the disability and death associated with chronic disease (9). However, clinical preventive services are substantially underutilized despite the human and economic burden of chronic diseases, the availability of evidence-based tools to prevent or ameliorate them, and the effectiveness of prevention strategies (9–11). For example, in 2015, only 8% of US adults aged 35 or older received all recommended, high-priority, appropriate clinical preventive services, and nearly 5% received none (12).

## Interview Study

It is far better to prevent disease than to treat people after they get sick (13). This is particularly true for chronic diseases, which are associated with suffering, large numbers of deaths, and high health care costs (2,7). Given the gap between the burden of chronic diseases and the utilization of preventive services, we set out to obtain from health care industry experts their perspectives on the levers and influencers that have the potential to increase utilization of clinical preventive care. The objective of our study was to gather experience-based insights that would be valuable to policy makers in developing strategies, programs, and partnerships across the health care industry to increase utilization of preventive services. We selected a qualitative interview study design for this investigation, which was conducted from December 2017 to June 2018. This project involved domain experts rather than human subjects as defined by 45 CFR part 46, and therefore institutional review board approval was not required.

### Recruitment of experts

We first identified experts with a background in working with decision makers in health care. We then narrowed our selection to 12 experts, each of whom had at least 10 years of experience in working with one or more types of organizations, including health systems, hospitals or physician groups, commercial payers, or state Medicaid agencies. We then conducted a short screening interview to confirm appropriate expertise and willingness to participate. After this initial selection process, we scheduled a 1-hour semistructured interview with each of 9 participants. Before beginning the interviews, the participants confirmed that they had no conflicts of interest that might bias their comments and that they would not disclose any confidential or proprietary information about the organizations for which they currently or previously worked. We tabulated details of their expertise (Table 1).

**Table 1:** Areas of Focus of Subject Matter Experts (N = 9) Participating in a Qualitative Interview Study Designed to Gather Information for Developing Strategies, Programs, and Partnerships Across the Health Care Industry to Increase Utilization of Preventive Services, 2018

Industry Sector	Role	Areas of Focus
**Payers**	Set payment models for preventive services or programs	Health plan collaborations with focus on value-based care transformation, population health, and consumerism Policies, processes, strategies, and information technology systems associated with successful Medicaid and Children’s Health Insurance Program programs, and other human services programs
**Health systems**	Develop and manage delivery of preventive services	Quality management for large health systems, including implementing health information technology and electronic health record transformations Strategy and operations effectiveness of health systems, including care management, vendor management, system design and implementation, post-merger integration, enterprise cost reduction Clinical transformation among health systems with focus on pay for performance and patient safety
**Providers and physicians**	Deliver or prescribe preventive services	Customer/patient experience strategies and digital transformation for health care providers Physician services design and implementation, including clinical integration, patient retention and physician loyalty, physician alignment, productivity and compensation, regulatory compliance, and ambulatory operations

### Interview questions

Increasing uptake of preventive services requires multifaceted strategies, including but not limited to organizational leadership, education, measurement, and reimbursement. With this in mind, we developed an interview guide (Table 2), which included a series of questions focused on how payers, health systems, and physicians determine their clinical and business priorities for resource allocation and quality improvement efforts. We asked about opportunities to include incentives for the use of preventive services under current and emerging designs of models for payment and delivery. We included questions about examples of successful implementation of preventive services strategies or models and about clinical–community linkages that focus on chronic disease prevention.

**Table 2:** Interview Questions Used in a Qualitative Interview Study Designed to Gather Information for Developing Strategies, Programs, and Partnerships Across the Health Care Industry to Increase Utilization of Preventive Services, 2018

Theme	Questions
Organizational leadership and decision making	How do health systems, payers, or providers determine their priorities (eg, deciding which strategies to focus on and what metrics to pay attention to, holding their physicians accountable for certain strategies, prioritizing certain interventions over others)? What are the primary drivers in the current health care delivery system – including both payment and delivery model designs – that shape guidelines, standards of care, or financial incentives?
Facilitators and barriers (measurement and reimbursement)	Could you describe facilitators and barriers that a typical health system faces when considering or implementing chronic disease prevention services? What additional opportunities (eg, performance measures, reimbursement structures) can be leveraged to drive uptake of prevention services among health system stakeholders? Under the current and emerging designs for models of payment and delivery, what are opportunities to better incentivize preventive services?
Successful models of prevention	Among the health systems you have worked with, are you familiar with successful implementation of preventive services, strategies, or models? Are you aware of any health systems that have implemented innovative community prevention programs or models that focus on chronic disease prevention?

Although primary prevention was not excluded, much of the discussion focused on secondary and tertiary prevention related to health care system interventions and community interventions linked to clinical services. Throughout the interviews, the participants were encouraged to draw from their experiences with organizations of various capacities and not to focus only on high-level performers or models that would be difficult for average organizations to adopt and replicate. Each interview was conducted via teleconference and facilitated by the first author (S.L.), a senior scientist with expertise in qualitative research methods.

## Interview Findings

Across all interviews, 4 findings emerged as major levers or influencers of preventive care. These findings cut across all health care industry sectors and organization types.

**Financial and economic considerations.** The most prominent theme was finances. All interviewees highlighted the importance of financial and economic considerations when organizations determine priorities and make decisions. These decisions include where to invest resources, what health benefits to cover, or how to bill for clinical services. In the words of one interviewee, “With no margin, there is no mission.”

**Use of metrics to drive change in the health care system.** The second finding was related to metrics and the importance of using metrics to drive change in the health care system. Interviewees stressed that measures continue to play a crucial role in the delivery of care, but the “right” metrics — outcome-focused, aligned across payers, and with sufficient financial incentives or risk — are needed to drive uptake of chronic disease preventive services. One participant, emphasizing that reporting and monitoring can drive change, noted, “Once external reporting is in place, measured outcomes are prioritized.” However, interviewees cautioned about the “metrics fatigue” that is plaguing health care providers, the misalignment of measures for reporting and quality ratings, and the current lack of financial risk for outcome measures associated with preventive care; in other words, payments to providers are not based on improvements in their patients’ health status.

**Role of health care payers.** The third finding focused on the role of health care payers (commercial payers/health plans, Medicaid, and particularly Medicare) in influencing uptake of preventive care services. Findings coalesced around the opportunities for payers to drive change in practice. As risk-bearing entities, they provide the payment models and the influence and incentives that can affect uptake of chronic disease preventive services. Several interviewees highlighted the importance of data for payers. As one expert explained, “Payers have the data that can often drive adoption or uptake of programs and interventions.”

**Rapid changes in health care reimbursement models.** The fourth finding focused on the pace of change in health care reimbursement models. The shift from volume-based reimbursement has been at the forefront of debate and discussion for years, but for typical health care delivery organizations, the transition to value-based reimbursement is still in early stages and is uneven across payers. As a result, the transition has not reached the “tipping point” for providers to change their practice patterns. As one interviewee observed, “There is some emphasis on value-based care, including focus on outcomes and reduced spending, but the view is generally short-term.” The health care industry will continue to move in the direction of value-based care, but changes in provider practice vary across systems and markets. There is also considerable room for continuing experimentation and evaluation to determine what reimbursement models work best and for whom.

## Discussion

Industry experts participating in this stakeholder interview process made it clear that most players in the health care system are aware of recommended preventive care services and understand the benefit of preventing disease for the patient and the larger health care system. Underutilization of preventive services is largely the result of an implementation gap rather than an information gap; in other words, providers do not prioritize preventive care services although they know that preventive services can reduce the incidence and burden of chronic diseases. A major reason the implementation gap exists is that financial incentives do not align with a focus on preventing chronic diseases. Currently, most providers, including hospitals and physicians, are paid to treat rather than to prevent disease. Payers have the potential to increase utilization of preventive services with value-based payment models and contractual requirements that include reporting on preventive health quality measures.

As the participants in our study offered their perspectives on the barriers and influences surrounding the coverage and delivery of preventive care services, much of the conversation focused on the influence of financial considerations on uptake of preventive care. However, participants generally agreed that financial incentives alone are unlikely to result in positive changes in the absence of a multipronged approach to increasing preventive services among people at risk of or living with chronic diseases. A multipronged approach would include strong organizational leadership, shifts in institutional culture, team-based care, systems of care that accommodate preventive services, and willingness of patients to seek out and engage in preventive care.
